# Asymmetric Male Mating Success in Lek-Breeding *Rhinella arenarum*

**DOI:** 10.3390/ani12233268

**Published:** 2022-11-24

**Authors:** Ulrich Sinsch, Katharina Hecht, Silvia Kost, Pablo R. Grenat, Adolfo L. Martino

**Affiliations:** 1 Institute of Integrated Sciences, Department of Biology, University of Koblenz-Landau, D-56070 Koblenz, Germany; 2 Ecología, Departamento de Ciencias Naturales, Facultad de Ciencias Exactas, Físico-Químicas y Naturales, Universidad Nacional de Río Cuarto, Ruta Nacional N° 36—km 601, Río Cuarto X5804BYA, Argentina; 3 Instituto de Ciencias de la Tierra, Biodiversidad y Sustentabilidad Ambiental (ICBIA), Universidad Nacional de Río Cuarto-CONICET, Río Cuarto X5804BYA, Argentina

**Keywords:** Anura, Bufonidae, reproductive behavior, advertisement call, phonotaxis, good genes as heterozygosity

## Abstract

**Simple Summary:**

Reproductive behavior is the result of tactics, which males employ to increase their success in mate acquisition, and of the attempt of females to choose those males, which increase the fitness and survival of their offspring. The aim of our study on pond-breeding neotropical toads (*Rhinella arenarum*) was to disentangle the interactions of male and female tactics in the mating system. Reproductive behavior took place in a lek, i.e., an assembly area where males advertised and defended suitable spawning habitats. Only one-third of the males present accounted for all matings observed as the result of two male tactics, namely calling and pre-mating fights to defend call positions to obtain access to unpaired females. In turn, females were selective, preferring mates of an adequate size for optimal fertilization success in size-assortative pair-formation. The lek organization of reproduction is unique in the *R. marina* species group, in which the interception of amplexed pairs is the predominant male tactic.

**Abstract:**

Mate choice is the attempt of an individual to gain higher reproductive fitness by preferring to mate with some individuals and not with others. We studied the role of mate choice in the mating system of the neotropical toad *Rhinella arenarum* by assessing male reproductive tactics for mate acquisition and the contribution of female choice for pair formation. In a shallow pond in central Argentina, we estimated male mating success and the corresponding reproductive tactics by focal observation. The variation of phenotypic and genotypic traits (size and shape, longevity, vocalization features, heterozygosity) was related to the observed mating success in 110 males. The phonotactic response of 21 reproductive females to conspecific advertisement call features was tested in arena experiments. Mating success was limited to 32 males, pair formation was size-assortative. The dominant reproductive tactics were advertising from call positions near suitable breeding sites and pre-mating fights with intruding males, whereas the interception of amplectant pairs and the displacement of mated males were never observed. Female phonotaxis was directed to conspecific choruses but complex and simple call structures were not distinguished. We conclude that the mating system is a lek combining pre-mating fights among males and female choice of slightly smaller males. Fights interfere with female choice, undermining size-assortative mating. This is a unique system in the *R. marina* species group, in which interception behavior dominates reproduction.

## 1. Introduction

In sexually reproducing vertebrates, mate choice is the attempt of an individual to gain higher reproductive fitness by exerting a preference to mate with some individuals and not with others [1,2]. Males as well as females have been shown to be selective in many taxa, and the outcome of male competition may influence female mate choice [3,4]. If choosiness depends on context-dependent phenotypic traits, lek mating systems may evolve [5,6]. In leks, males typically occupy and defend small areas in which they vocalize [7]. The call positions are defended in physical interactions if intruders of the same sex approach too closely. Females choose their mates by moving both within and between aggregations of males [7]. If selectivity leads to a genetically based improved fitness, sexual selection may promote mate choice [8,9]. For example, the good genes theory predicts that males bear condition-dependent traits that indicate genetic qualities, and that females prefer males carrying genes that will improve offspring fitness [10,11]. A modification of this theory, the good genes as heterozygosity hypothesis, predicts that female choice will favor more heterozygous mates generating more heterozygous offspring, which will enjoy greater fitness due to an increased allelic diversity [12,13,14]. Empirical support of this hypothesis remains controversial in vertebrate taxa [14,15,16,17,18,19,20].

Anuran amphibians provide a variety of examples, in which male mating success varies dramatically within leks or populations (reviews: [21,22]). A direct observation of mating behavior in *Hyla chrysoscelis* showed that 35% of males accounted for all pair formations observed [23]. Genetic estimates of effective population size suggest that only 1–17% of males in toad populations account for successful fertilizations (*Bufo bufo*: [24]; *Epidalea calamita*: [25]). Asymmetrical male mating success may arise from scramble competition as well as from female choice. So far, no genetically based strategies have been identified in anurans and, therefore, distinct reproductive phenotypes in breeding assemblages employ most likely alternative reproductive tactics within condition-dependent strategies [26]. There are at least three alternative tactics adopted by males to increase an individual’s chance of mate acquisition: intensive and elaborate acoustic advertisement, silent satellite behavior, or aggressive interception of already formed pairs [27]. In the lek-breeding frog *Ololygon rubra,* male mating tactics considerably affect female choice and thus, the outcome of pair formation (amplexus; [28,29]). Almost all amplexi (93%) resulted from female choice favoring males that were about 80% of the female’s size, whereas silent satellite males accounted for the remaining 7%. Yet, large males circumvented the initial female choice in 78% of the pair formations by displacing smaller males. Thus, size-assortative mating can indicate female choice in the species with sedentary calling behavior but also male–male competition and their combination [30].

Mating systems of neotropical toads (genus *Rhinella*) vary considerably among species and may include lek-chorusing in ponds, terrestrial advertisement, antiphonal call alternation between neighboring males, aggressive male–male interactions, prolonged aggressive female–female encounters, and female choice based on advertisement call features [31,32,33,34]. The reproductive behavior of the widespread common toad *Rhinella arenarum* is almost unknown, except for pond breeding associated with intensive male advertisement and size-assortative mating, indicative of female choice [35]. Preliminary observations in central Argentina suggested the presence of a lek system (di Tada, Martino, Sinsch, unpubl. observ.). In this study, we explored male mating tactics, males’ phenotypic and genetic traits associated with mating success, and female choice to unravel the potential interactions leading to size-assortative mating. We tested three predictions derived from the theory of sexual selection in reproductive behavior: (1) the mating success differs among males and is related to phenotypic traits (morphology, age, communication); (2) heterozygous males enjoy mating success more frequently than homozygous ones; (3) female choice is based on acoustic signals of males (advertisement call features).

## 2. Materials and Methods

### 2.1. Study Species

**Figure 1:**
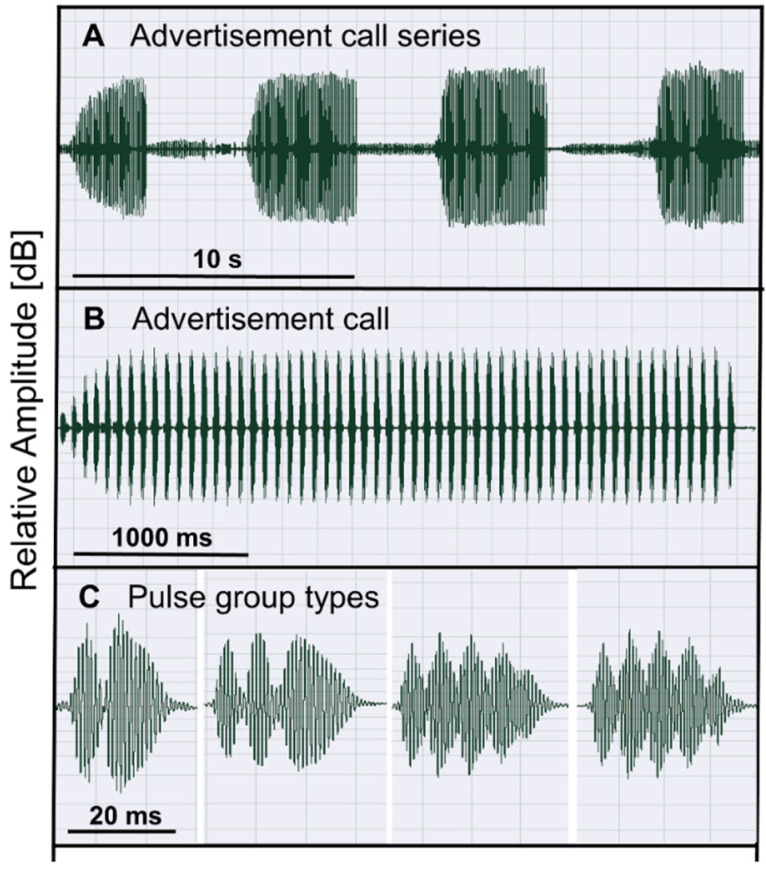
Oscillograms of the advertisement call of *R. arenarum*. (**A**) Series of four consecutive calls. (**B**) Single call consisting of 56 pulse groups, each formed by 2 to 5 pulses. (**C**) Close-up of the pulse group types. Bars indicate temporal resolution. The call series was recorded at 20.9 °C.

In Argentina, Bolivia, and Brazil, the toads *R. arenarum* are abundant in dry, open habitats that provide small ponds or bogs with stagnant water [36,37]. They are tolerant to anthropogenic disturbance. During the prolonged reproductive season (September to January), males call at day and night from the shore or in the shallow water to attract females [36]. Oviposition in these waterbodies occurs in discrete boosts, mostly in October and November, following rainfall. Vocal communication of males includes advertisement and release calls [38,39]. Advertisement calls of *R. arenarum* are given in series. A single call consists of several pulse groups, in which the number of pulses per pulse group may vary from 2 to 5 (Figure 1).

### 2.2. Study Site

Surveys of male reproductive behavior and female phonotaxis were performed at the campus of the Universidad Nacional de Rio Cuarto (Rio Cuarto, Cordoba province, Argentina), which included a permanent pond (“Charca de las brujas”; 4500 m^2^ surface area, 33°06′42″ S; 64°18′15″ W, 428 masl). We surveyed the local population of *R. arenarum* during 33 nights of the breeding period of the year 2000 (10 October–8 December, seasonal temperature and rainfall variation shown in Supplementary Figure S1) and collected 110 males for the study of phenotypic and genotypic traits. During all monitoring nights, the mating success of each male was classified into successful (males found at least once in amplexus) or unsuccessful (advertising males that were never found in amplexus). Advertisement call series were recorded of as many males as possible at their natural calling positions within the pond (water temperature range: 16.3–28.7 °C) by positioning the microphone within 10 cm of the calling male. The recording device included a portable tape recorder (Uher, Bad Homburg, Germany, 4000 S) and a condenser microphone (Sennheiser, Wedemark, Germany, Type MKH 415 T). Then, all males present at the breeding pond were hand-captured, transported to the nearby laboratory, tagged individually with passive integrated transponders (PITs) and released again in the pond during the following day. Recaptured males were identified by their unique PIT code and released again following each collection survey the same night. All individuals were toe-clipped (3rd finger of right hand) for later skeletochronological age estimation before release. Additionally, we collected blood samples (60 µL) by puncture of the vena angularis [40] using heparinized capillaries for the assessment of the individual allozyme patterns and heterozygosity.

Phonotactic responses of females to standardized male advertisement calls were tested in arena experiments at the university campus about 500 m from the breeding pond to avoid interference with the natural soundscape. We collected 38 females from the same population as males during the breeding period of 2015 (29 September–25 November, seasonal temperature and rainfall variation shown in Supplementary Figure S1). Females were hand-captured at 12 am–2 pm, if they were found in amplexus, but before the onset of spawning. Pairs were kept singly in sound-proof plastic boxes in 5 cm deep water. Phonotactic trials were performed immediately following the capture survey, i.e., from 2 pm to 6 pm. Unfortunately, 17 pairs started spawning in the boxes, so they were excluded from the experimental trials. Snout–vent length (SVL) of these females and males was measured to test for size-assortative mating. Immediately before the arena trials in the afternoon of the same day, the remaining 21 females were separated from the amplecting male.

We adhered to the ASAB/ABS Guidelines for the Use of Animals in Research. The experiments complied with current legislation in Argentina. The animal study protocol was approved by the Ethics Committee of the National University of Rio Cuarto-COEDI, UNRC (Protocol Code 241-01; date of approval: 24 October 2000). The toads were kept in the laboratory for less than 1 day and then released into their natural environment.

### 2.3. Male Traits

We analyzed four traits that may be subject to selection by female choice: size and shape attributes, longevity, advertisement call features, and genetic constitution (heterozygosity in enzymes of the intermediate metabolism).

Morphometric features: Thirteen morphometric variables of each male were measured with a caliper (SOMET Inox extra/MAHR 16 ES) to the nearest 0.1 mm. These measures were: (1) Snout–vent length (SVL), (2) head width (HW), (3) snout–eye distance (ED), (4) eye–nostril distance (END), (5) inter nares distance (IND), (6) humerus length (HL), (7) radius length (RL), (8) hand length (HND), (9) length of the 3rd finger (F3L), (10) femur length (FL), (11) tibia length (TL), (12) foot length (FOL), and (13) length of the 4th toe (T4L). All measurements were taken by S.K.

Longevity: The age of each male was assessed skeletochronologically using standard procedures [41,42]. Phalange bones obtained by toe-clipping were preserved in 70% ethanol until histological processing. The diaphysis of each phalanx was cross-sectioned at 8–10 μm using a JUNG RM2055 and stained with 0.05% cresyl violet for the light microscopic count of the number of lines of arrested growth (=LAG) using an Olympus BX 50. The number of LAGs was counted in the periosteal part of the bone independently by two authors (S.K., U.S.).

Advertisement call features: Oscillograms of the recorded call series were generated with a Medav Mosip 3000 Signal Processing System. For each call series, we analyzed five calls (range: 2–8) on average, resulting in 50 calls from successful males and 155 calls from unsuccessful ones. The variables measured in each advertisement call were: (1) call duration (ms); (2) number of pulse groups per call (n); (3) repetition rate of pulse groups (n per s); (4) number of different pulse group types (2–5 pulses per pulse group) per call; (5) number of pulses per call (n); (6) pulse rate (n per s); (7) dominant frequency (Hz). The corresponding SVL of the male and the water temperature at its calling site complemented the data on each call.

Allozyme pattern/heterozygosity: Blood samples (60 µL) of each male used for genotype assessment were dissolved in 300 µL homogenate buffer (Tris-EDTA-NADP at pH 7.0) and stored at −65 °C until use. Sample aliquots of 0.3–3 μL blood homogenate were applied to commercial cellulose acetate plates (PHERO-cel, 5.7 × 14.0 cm) and submitted to a continuous horizontal electrophoresis [43]. Buffer systems and duration of electrophoresis were 30–40 min at room temperature: (1) Tris-Glycine at pH 8.5 and constant 200 V; (2) CAAPM (citric acid aminopropyl morpholine) at pH 7.0 and constant 130 V. Following electrophoresis, each gel was stained using standard recipes (Murphy et al., 1996). The allozyme pattern of blood consisted of eight enzyme systems controlled by eight presumptive loci: alcohol dehydrogenase (ADH, EC 1.1.1.1), fumarase (FUM, 4.2.1.2), glucose-6-phosphate isomerase (GPI, 5.3.1.9), glucose-6-phosphate dehydrogenase (G6PD, 1.1.1.49), malate dehydrogenase (MDH, 1.1.1.37), malic enzyme (ME, 1.1.1.40), mannose-6-phosphate isomerase (MPI, 5.3.1.8), phosphoglucomutase (PGM, 2.7.5.1). Electromorphs (presumptive alleles) of each locus were designated alphabetically from cathode to anode. We verified the individual allozyme patterns for heterozygotes in polymorphic loci.

### 2.4. Female Response to Male Calls

The phonotactic response of reproductive females to standardized advertisement calls was tested in an ellipsoid arena (ca. 1.3–1.5 m radius) placed on plain ground and shaded by a tree group. The arena ground was soil covered with short-cut grass. The arena walls were made of black cotton 40 cm in height that was fitted vertically onto wood sticks. Two loudspeakers (Logitech z120 Stereo Speakers) were placed outside the wall 45° left and right of the north–south axis. Signal type and amplitude were controlled by a Fujitsu Lifebook E544. Average sound level of all test call series was 80 dB, simulating a male calling in about 1 m distance from the center of the arena as indicated by empirical measurements of sound pressure level in the natural environment. If a single call type was tested, only one loudspeaker was active, whereas combinations of distinct call types were always given simultaneously from the two loudspeakers.

Based on the advertisement call features of males with and without reproductive success, we synthesized four types of standardized call series (duration of 5 min each) that differed in the inter-call interval, the number of pulse groups per call, and the number of pulses per pulse group, i.e., the attributes associated with reproductive success. Pulse groups were copied from field-recorded calls. We chose pulse groups with a dominant frequency of 1308–1386 Hz. Amplitude modulation of the artificial calls mimicked that of a natural call with ten pulse groups of gradually rising amplitude followed by pulse groups of maximum amplitude until the end of the call. Type 1 and 2 calls consisted exclusively of pulse groups that included five pulses. Type 1 calls were longer than type 2 calls (16.1 s vs. 10.2 s), had shorter inter-call intervals (4.0 s vs. 7.9 s), and the pulse group was shorter (33 ms vs. 39 ms). Both call types represent optimized modifications of structural call features exclusive to successful males (see results), assuming that attractiveness to females was above average. Call series type 3 consisted of short calls (duration: 4.9 s) that had pulse groups with two and three pulses per pulse group (duration: 22–23 ms). Type 3 calls were the most frequent call type in the field and given by males with and without reproductive success (see results). Call series type 4 consisted exclusively of very short calls (duration: 1.5 s) with two pulses per pulse group (duration: 15 ms). In the field, type 4 calls were emitted rarely by males with and without reproductive success and assumed to have the lowest attractiveness (see results). The energetic investment in calling mimicked by these call series decreased from type 1 to type 4, with type 3 representing an average investment of a calling *R. arenarum* male.

Experimental analysis of phonotactic response to test call series started by placing a female into the arena below an opaque plastic bowl for one minute to reduce handling stress. The distance to each loudspeaker at the external arena wall was about 90 cm. Following the first minute, the test call series was played for 30 s, with the female still sitting below the bowl to allow for a preliminary neural processing of the call. Then, the bowl was manually lifted and the female’s movement in the presence of the test calls recorded for the next three minutes with a Panasonic Lumix DMC-FZ200 DigiCam. Each female was tested four times consecutively in two randomly chosen experimental set-ups out of the three described above. The same call types were tested in two trials, with calls played from inverted loudspeaker positions.

Experiment 1 was designed to test for a phonotactic orientation toward a standardized call of average attractiveness. We played call type 3 from a single loudspeaker, but at inverted positions in the two consecutive trials. Experiment 2 aimed to test whether females can distinguish the small differences between calls including features exclusive to successful males. We used call types 1 and 2, played simultaneously from the two loudspeakers. The position of each call type was inverted in the two consecutive trials. Experiment 3 gave females the choice between calls of high and low energetic investment. We used call types 1 and 4, again played simultaneously, and with positions inverted in the two consecutive trials. Females were released at the breeding pond immediately following experimental tests.

Variables quantified in each trial were: (1) latency: time (s) until the first movement from the start position; (2) movement to arena wall: time (s) between first movement and the first contact with the arena wall; (3) initial orientation: direction (in 5° classes) of first contact with the wall relative to geographic north (0°); (4) movement to loudspeaker: time (s) from first movement to reaching one of the loudspeakers; (5) final orientation: choice of call 1 or of call 2 or no choice within three minutes.

### 2.5. Statistical Analyses

Descriptive statistics are given as the arithmetic mean ± standard error, mostly of the log10-normalized variables. Statistical comparison of variables was conducted using an analysis of co-variance (ANCOVA), with SVL as continuous covariable in the morphometric data set and with air temperature in the bioacoustic data set. We fitted regression models to the relationships between female and male SVL and dominant frequency of the advertisement call and male SVL, to test for size-assortative mating and for bioacoustic size information, respectively. For the age distribution, we chose the median for description of the skewed distribution and the Kolmogorov–Smirnov test to compare distributions. A discriminant analysis (procedure: backward selection at F = 4.0) was run on the morphometric and bioacoustic data sets to distinguish between the morphology and call structure of successful and unsuccessful males. The significance level used was *p* < 0.05. Tests were performed using the statistical package STAGRAPHICS Centurion XVIII (Statpoint, Inc., Warrenton, VA, USA, 2018).

## 3. Results

### 3.1. Reproductive Behavior at the Study Pond

**Figure 2:**
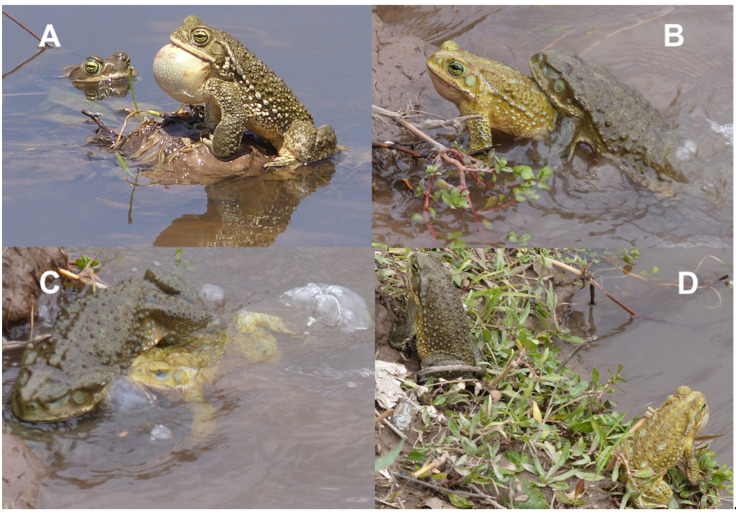
Male–male encounter of breeding *R. arenarum*. (**A**) Advertising male at a typical call site. (**B**) Early stage of aggressive encounter, pushing the rival with the forelimbs. (**C**) Late stage of aggressive encounter, pushing the rival with the hindlimbs below water surface. (**D**) Final stage of aggressive encounter, spacing at new call positions. All photographs by K. Hecht.

The main calling activity took place between end of September and end of November, but female presence at the pond was limited to 6–9 days (Supplementary Figure S1). The males’ calling activity peaked around midday (11 am–2 pm) and during the evening (6 pm onward). Groups of 10–30 males took ephemeral call positions in the shallow part of the pond, typically sitting upon plant material or soil heaps (Figure 2A). Individuals were usually spaced 0.5–1.3 m from each other. Males switched between calling and silence, but there were no permanently silent satellites. Females approached the local choruses, stopped at about 50 cm from a calling male/male group, and observed the local males. They were clasped as soon as one or more males noted the female’s presence. Reproductive behavior included two types of aggressive male–male encounters. The most frequent type was the attempt to displace a local male from its call position, independent from the presence of females. The intruding male clasped the local male with the forelimbs attempting to push it into the water (Figure 2B). If successful, the intruding male positioned itself upon the back of the local male and used its hindlimbs to push it further away from the calling position into the open water (Figure 2C). At the end of such an encounter, the two males returned to a new or former call position at least 20 cm apart from each other (Figure 2D). The less frequent type of aggressive male–male interaction occurred when a female approached a group of callers. Males fought using fore- and occasionally hindlimbs to get into a position to clasp the female. Following successful amplexus, attacks ceased. We never witnessed interference of single males with pairs in amplexus or during oviposition. The close vicinity of the former calling site of the paired male was often later the spawning site.

Female and male SVL of amplecting pairs correlated significantly (*p* = 0.0304, R^2^ = 27.6%, N = 17; best fit: double squared regression model) confirming the earlier report on size-assortative mating (*p* < 0.001, R^2^ = 46.6%, N = 20) by Bionda et al. [35]. The SVL range of females was 97–125 mm, and that of males was 85–122 mm. Males in amplexus were about 20% smaller than the corresponding females.

Mating success was asymmetrical among males, with only 32 out of the 110 studied males observed in an amplexus at least once. The individual number of amplexi was four for one male, whereas one amplexus was recorded for the remaining 31 successful males. The 78 males classified as unsuccessful were likely a heterogenous group because some of them may have had an unnoticed amplexus, specifically during the midday peak of calling activity. We obtained morphometric data and allozyme patterns from all males, age estimates from 105 individuals (31 successful, 74 unsuccessful), and advertisement calls from 46 individuals (11 successful, 35 unsuccessful).

### 3.2. Traits of Males with and without Mating Success

We found significant differences between successful and unsuccessful males with respect to morphometric, demographic, and bioacoustics traits. Most morphometric parameters did not differ between the groups, but the humeri (HL) and third finger (F3L) were significantly longer in successful males (Table S1). The discriminant analysis (procedure: backward selection) condensed the log10-normalized morphometric parameters into three (SVL, HL, F3L), which significantly contributed to the separation of the groups. The standardized discriminant function (eigenvalue = 0.126, Wilks Lambda = 0.888, χ^2^ = 12.63, df = 3, *p* = 0.0055) was DF_morph_ = −1.572 × log10(SVL) + 1.537 × log10(HL) + 0.774 × log10(F3L). The classification success of DF_morph_ was 71.9% in successful males and 65.4% in unsuccessful ones (a priori probability 50%). The main distinctive feature was the humerus/SVL ratio (average ± SE: 0.3443 ± 0.0023 vs. 0.3378 ± 0.0015) that was significantly larger in the successful males (ANOVA, F_1,109_ = 5.61, *p* = 0.0196). At the same body length, successful males had longer and stouter arms than unsuccessful ones.

The genetic variability within the eight loci scored was low in the study population. Six loci were monomorphic, one (PGM) had two alleles with a strong dominance of allele A (0.97), and one (PGI) had four alleles (A: 0.20; B: 0.54; C: 0.23; D: 0.03). The GPI allele frequencies did not differ between the two male groups, but successful males were less heterozygous than unsuccessful ones (50% vs. 63%).

**Figure 3:**
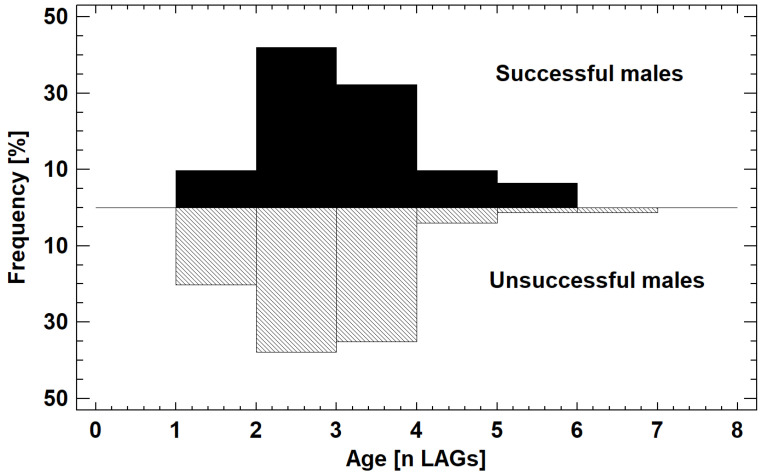
Age structure of male breeding *R. arenarum*. For statistical details see text.

The age structure of the two male groups did not differ with respect to medians (2 LAGs), but significantly in the shape of distribution (Kolmogorov–Smirnov-test, *p* = 0.00007; Figure 3). This was because young males (age class 1 LAG) were more frequent in the unsuccessful group (25% vs. 10%), and old males (age classes > 3 LAGs) in the successful group (17% vs. 7%).

**Figure 4:**
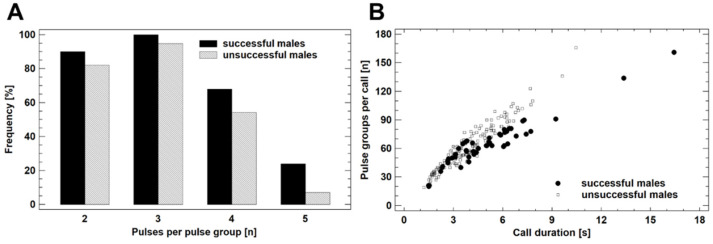
Advertisement call features of breeding *R. arenarum*. (**A**) Frequency of pulse group types in the advertisement calls (numbers do not add up to 100 because calls included usually more than one pulse group type). (**B**) The most distinctive call variables according to the discriminant analyses. For details see text.

The dominant frequency of the advertisement calls was negatively correlated with the size of the caller (*p* < 0.0001, R^2^ = 21.8%, N = 162; best fit: reciprocal Y, squared X regression model). The most significant differences between the groups concerned pulse group structure, namely pulse group duration and inter-pulse group intervals (Table S2). The average complexity of calls (number of different pulse group types present in a call) did not differ between the two groups (2.5 pulse group types), if adjusted for ambient temperature (ANCOVA, F_1,204_ = 1.80, *p* = 0.1870). Yet, the calls of the successful males included more often pulse groups with five pulses (24.0% vs. 7.1%) and more often calls including four pulse group types (24% vs. 5.1%) than those of unsuccessful males (Figure 4A). The discriminant analysis (procedure: backward selection) condensed the call parameters into three, which significantly contributed to the separation of the groups (Figure 4B). The standardized discriminant function (eigenvalue = 0.366, Wilks Lambda = 0.732, χ^2^ = 62.91, df = 3, *p* < 0.00001) was DF_call_ = −23.6358 + 0.00247 × (call duration) − 0.1092 × (pulse groups per call) + 0.9059 × (pulse rate). The classification success of DF_call_ was 66.0% in successful males and 83.2% in unsuccessful ones (a priori probability 50%). In synthesis, successful males produced calls with less, but more complex pulse groups, which had a greater duration and longer inter-pulse group intervals.

### 3.3. Female Response to Male Calls

All but one female tested in the arena set-up responded to the calls by leaving the start position toward the arena wall. The log10-normalized latency to start moving varied significantly among the 21 individuals (three-factor ANOVA, F_20,82_ = 3.25, *p* = 0.0002; range of least square means: 0.75–60.4 s) and between trials with one or two call types displayed simultaneously (three-factor ANOVA, F_1,82_ = 4.98, *p* = 0.025; 21.4 ± 5.3 s vs. 14.6 ± 2.6 s), but not with respect to the order of the tests performed (three-factor ANOVA, F_3,82_ = 1.72, *p* = 0.1727). With respect to the duration of initial orientation (log10-normalized time until the first contact with the arena wall), the significant inter-individual differences persisted (three-factor ANOVA, F_20,82_ = 3.04, *p* = 0.0005; range of least square means: 3.2–71.0 s), whereas the number of active loudspeakers did not significantly influence the response time (three-factor ANOVA, F_1,82_ = 0.01, *p* = 0.9299). In 7 of the 83 trials in which females moved, they did not reach a loudspeaker position within the 3 min time limit. None of the three considered factors affected the time taken for a final directional choice, i.e., the position of a loudspeaker (three-factor ANOVA, *p* > 0.05).

**Figure 5:**
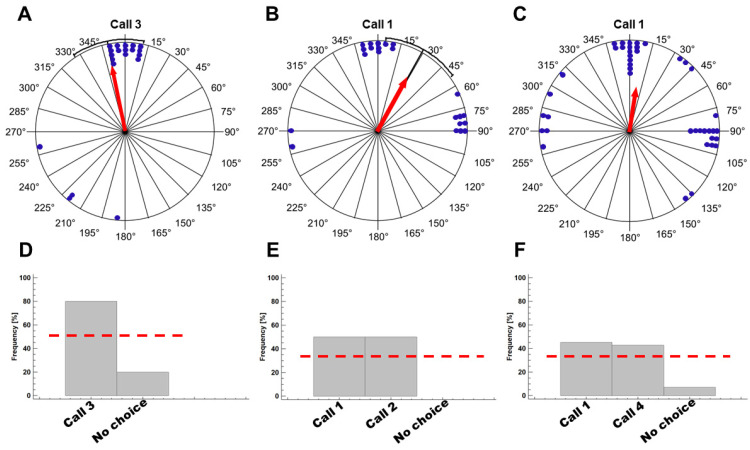
Phonotactic orientation of reproductive *R. arenarum* females. (**A**–**C**) Initial orientation. The circular distribution of females’ directional choice (blue dots) is standardized to a loudspeaker position of 0°. Significant mean vectors are shown as red arrows. (**D**–**F**) Final orientation. Grey columns give the frequency of directional decisions, dashed red line the expected frequency of a chance decision among loudspeaker positions and no choice. Details are given in the text.

In experiment 1 (test for the presence of phonotaxis, series of type 3 calls displayed by one loudspeaker), the initial orientation was directed toward the loudspeaker position with an accuracy of ±10° (Figure 5A). The mean vector length (0.669) differed significantly from zero (Rayleigh test, z = 8.96, *p* < 0.0001). The mean direction was 349.1° with the corresponding confidence interval, including the correct direction. The final directional choice was, in 80% of the trials, the loudspeaker position, significantly deviating from the a priori probability of 50% (Kolmogorov–Smirnov test, *p* < 0.0001; Figure 5D). One female failed in the two trials, two females in their first trial.

In experiment 2 (choice between series of type 1 and type 2 calls), the females oriented significantly toward one of the two loudspeaker positions, yielding a bimodal circular distribution (Figure 5B). If the position of the loudspeaker displaying the call 1 was set to 0°, the resulting mean vector length (0.617) differed significantly from zero (Rayleigh test, z = 7.81, *p* < 0.0001). The mean direction was 29°, but the corresponding confidence interval did not include any of the loudspeaker positions due to the bimodal distribution. The final directional choice was, in 50% of the trials, the call 1 position, and in the other 50% it was the call 2 position, in agreement with a random choice (Figure 5E). One female consistently chose the type 1 call, while another one the type 2 call, and nine changed their preference between the two consecutive tests.

In experiment 3 (choice between series of type 1 and type 4 calls), the initial orientation was like that in experiment 2, i.e., females moved significantly toward one of the two loudspeaker positions, yielding a bimodal circular distribution (Figure 5C). If the position of the loudspeaker displaying the call 1 was set to 0°, the resulting mean vector length (0.485) differed significantly from zero (Rayleigh test, z = 5.48, *p* < 0.001). The mean direction was 5°. The final directional choice was, in 45.2% of the trials, the call 1 position, while in the other 42.9% it was the call 4 position (Figure 5F). Three females consistently chose the type 1 call, three females the type 4 call, and 12 changed their preference between the two consecutive tests. Another three females refused to approach any of the signals.

## 4. Discussion

We provide evidence that *R. arenarum* males display typical lek breeding behavior, confirming earlier anecdotal observations. The pre-mating and mating behavior included elaborate male acoustic advertisement, male fights for calling positions, female phonotactic approach to calling males, male–male aggressive interactions before clasping a nearby female, pair formation with the winning male, and oviposition in the shallow water near the calling site. Surprisingly, the males never intercepted pairs in amplexus to displace the mating male, the dominant reproductive tactic in *R. horribilis* and *R. marina*, which are closely related to *R. arenarum* [44,45,46,47]. Instead, the dominant reproductive male tactics were to fight for call sites and for approaching females, i.e., pre-mating fights, which effectively limited mate acquisition to few males of the local lek. This unusual male mating tactic has evolved at least twice in the genus *Rhinella*, in *R. arenarum* pertaining to the *R. marina* group, and in *R. ornata* pertaining to the *R. crucifer* group, the sister clade of the *R. marina* group [34,45]. Consequently, we focus the discussion on the phenotypic and genotypic traits that were associated with successful mate acquisition without pair interception (predictions 1 and 2).

Unlike pair formation in *R. marina*, our study corroborates the trend to size-assortative mating in *R. arenarum*, but less pronounced as reported in Bionda et al. [35,45,46]. Still, the preference for males that are about 20% smaller than the female was evident and suggests a significant contribution of female choice to the mating system [30,48,49]. As the correlation between female and male SVL was weak, we further discuss the potential mechanisms undermining a more precise female choice (prediction 3).

### 4.1. Prediction 1: Mating Success Differs among Males and Is Related to Phenotypic Traits

Regardless of whether mate acquisition is mainly the result of scramble competition among males or female choice, we expected males amplecting females to differ in one or more phenotypic traits from those without successful mate acquisition. As predicted, we found several distinguishing traits in successful males, such as stouter forearm morphology, more elaborate advertisement calls, and to a lesser extent greater age.

The subtle morphological differences related to fighting ability and the absence of a bias in general size (SVL) toward successful males emphasize that aggressive male behavior is not directed to the interception of a pair in amplexus and to the replacement a smaller mated male, a tactic practiced by other lek breeders [28,29,50]. Aggressive male–male encounters rather represent a territorial behavior within a lek employed to temporarily defend call positions near a suitable spawning site, a tactic also observed in *R. ornata* and in *Lithobates catesbeianus* [34,48]. Experienced (i.e., older) males appear to outcompete recently matured males (age class 1 LAG) that also tend to be smaller than most older males [42]. Again, the role of experience/age in mating success resembles the lekking behavior of *L. catesbeianus* [48].

The acoustic signaling of males had at least two properties providing information on approximate body size and the ability to invest energy in breeding behavior at a distance to conspecific females and males. As in many other toad species, size information is coded in the dominant frequency of the advertisement call, allowing for size-assortative mating of choosing females and the avoidance of aggressive encounters between males of different sizes [20,32,51]. Yet, the transmitted information was not precise (low R^2^ of frequency–size relationship) and probably needed evaluation in close-up encounters. Mating success was related to the ability to produce longer and more complex advertisement calls, an indicator that these males could spend more energy in the breeding behavior than callers with more simple calls. Traits in acoustic signaling may indicate good genes and promote sexual selection by female choice, as demonstrated in the lek-chorusing *H. intermedia* and *Zhangixalus prasinatus* [18,20].

In summary, correlated morphological and acoustic traits distinguish the phenotypes of males with and without mating success and allow for female choice before pair formation. It remains open whether these traits are inherited (good genes model of sexual selection) or vary depending on context.

### 4.2. Prediction 2: Heterozygous Males Enjoy Mating Success More Frequently Than Homozygous Ones

The ultimate reason for the evolution of female choice is to increase the fitness of the offspring by choosing males which carry good genes [10,11]. The good genes as heterozygosity hypothesis predicts that heterozygous individuals have fitness advantages over more homozygous ones, and are therefore a better choice for mating [1]. We did not find support for this hypothesis in *R. arenarum* because successful males included less heterozygous genotypes than unsuccessful ones. The same was true for red-backed toadlets *Pseudophryne coriacea*, in which females preferred related rather than heterozygous males [19]. Thus, the limited evidence suggests that the good genes as heterozygosity hypothesis should be rejected for anurans.

### 4.3. Prediction 3: Female Choice Is Based on Acoustic Signaling of Males

Phonotaxis toward male advertisement calls is a common feature of anuran females (and males) and is used to approach a breeding chorus from larger distances, e.g., [32,52,53]. In lek-chorusing *H. versicolor,* females prefer choruses of several males over single caller advertisement [54]. Our phonotaxis experiments with female *R. arenarum* suggest a similar preference because the motivation to start moving toward calls was greater (shorter latency) when displaying two callers rather than a single caller. In fact, our field observations corroborated the fact that females moved toward a group of males with neighboring call positions and stopped before reaching a specific male, suggesting that the chorus was the cue for the approach, and not individual call features.

Two choice experiments at short distances indeed did not reveal any preference for more elaborate advertisement calls typical for successful males in leks. As we tested calls with about the same dominant frequency (weak indicator of male size), we cannot exclude that the decisive feature for choosing a specific male was dominant frequency instead of elaborate call structure. This has been shown in *R. marina* females, which tended to choose calls that indicated a male size, which was slightly smaller than themselves [32]. The weak reliability of the size information coded in the dominant frequency in *R. arenarum* may explain the low but significant correlation between female and male size. The male winning the male–male fighting contest near an unpaired female was the mate in pair formation, not necessarily the perfect size match. This behavior probably contributed most to confounding male size.

## 5. Conclusions

The mating system of *R. arenarum* is a lek, combining aggressive interactions between males in the defense of attractive calling and spawning sites and for the acquisition of unpaired females and the female preference of slightly smaller males as optimal mates. This male reproductive tactic is unique in the *R. marina* group, but was also reported for *R. ornata,* pertaining to the sister clade [34,47]. Pre-mating male fights and female choice interfere with each other, interfering with size-assortative mating in this species without preventing female mate choice based on male size completely. Thus, the asymmetrical mating success *R. arenarum* results from the pre-mating scramble competition and female preference for 20% smaller-sized males. This mating system contrasts sharply from that of closely related *Rhinella* toads, in which few males dominate mate acquisition by displacing smaller, already mated males due to their larger size, rendering female choice unfeasible.

## Data Availability

All data generated or analyzed during this study are included in this published article (and its Supplementary Information Files).

## Supplementary Materials

The following supporting information can be downloaded at: https://www.mdpi.com/article/10.3390/ani12233268/s1, Figure S1: Seasonal temperature and rainfall variation; Table S1: Morphometric features of successful and unsuccessful males; Table S2: Bioacoustic features of successful and unsuccessful males.
