# Supplementary material for: Asymmetric Male Mating Success in Lek-Breeding Rhinella arenarum
Source: Animals (Basel). 2022 Nov 24;12(23):3268. doi: 10.3390/ani12233268 (PMC9740229; doi:10.3390/ani12233268)

animals

Figure S1. Seasonal temperature and rainfall variation at the study pond "Charca de las brujas" (4500 m² surface area, 33°06'42'' S; 64°18'15'' W, 428 m a.s.l.), located at the campus of the Universidad Nacional de Rio Cuarto (Rio Cuarto, Cordoba province, Argentina).

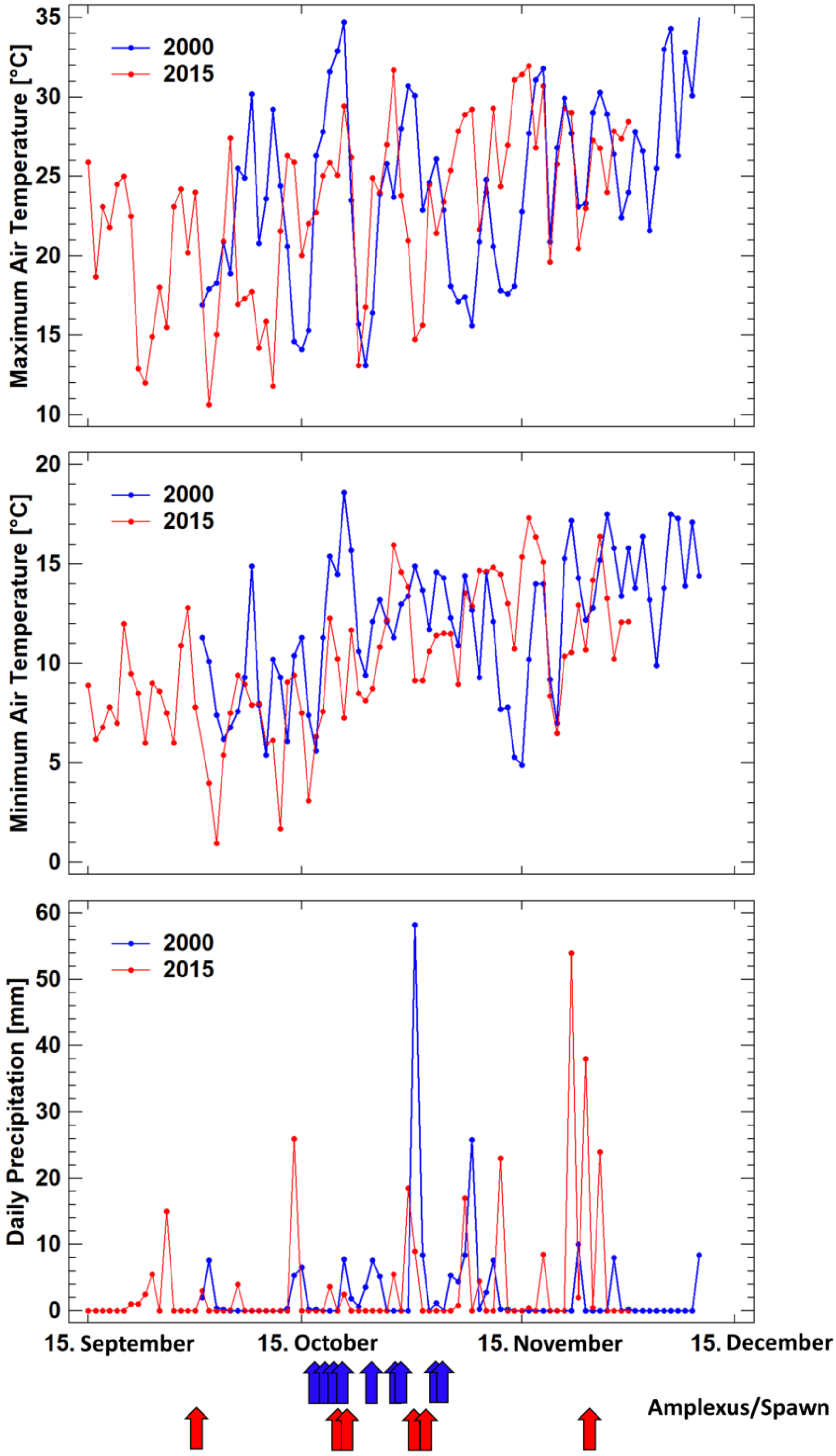

Supplement: Supplementary file 1 [file animals-12-03268-s001.zip › Sinsch et al. 2022 supplementary figure 1.pdf]
