# Supplementary material for: Asymmetric Male Mating Success in Lek-Breeding Rhinella arenarum
Source: Animals (Basel). 2022 Nov 24;12(23):3268. doi: 10.3390/ani12233268 (PMC9740229; doi:10.3390/ani12233268)
Supplement: Supplementary file 1 [file animals-12-03268-s001.zip › Sinsch et al. 2022 supplementary table 1.pdf]

animals

MDPI

Table S1. Between-group variation of log10-normalized morphometric parameters in males with and without mating success. Groups were compared using an ANCOVA with SVL as a covariate. Data are given as least square means and corresponding standard error.

Morphometric parameter	Successful males (N=32)	Unsuccessful males (N=78)	Significance P
Snout-vent length (SVL)	1.9995±0.0068	1.9938±0.0044	0.4841
head width (HW)	1.5183±0.0027	1.5165±0.0017	0.5746
snout-eye distance (ED)	1.3982±0.0026	1.3972±0.0016	0.7730
eye-nostril distance (END)	1.2993±0.0033	1.2978±0.0021	0.6967
internare distance (IND)	0.8683±0.0047	0.8641±0.0030	0.4536
humerus length (HL)	1.5329±0.0030	1.5235±0.0019	**0.0083**
radius length (RL)	1.4333±0.0043	1.4241±0.0028	0.0730
hand length (HND),	1.3355±0.0041	1.3272±0.0026	0.0872
length 3rd finger (F3L)	0.9444±0.0075	0.9201±0.0048	**0.0075**
femur length (FL)	1.6168±0.0043	1.6113±0.0028	0.2870
tibia length (TL)	1.5989±0.0029	1.5932±0.0019	0.1036
foot length (FOL)	1.7653±0.0039	1.7639±0.0025	0.7613
length 4th toe (T4L)	1.5682±0.0039	1.5624±0.0025	0.2227
