# Supplementary material for: Asymmetric Male Mating Success in Lek-Breeding Rhinella arenarum
Source: Animals (Basel). 2022 Nov 24;12(23):3268. doi: 10.3390/ani12233268 (PMC9740229; doi:10.3390/ani12233268)
Supplement: Supplementary file 1 [file animals-12-03268-s001.zip › Sinsch et al. 2022 supplementary table 2.pdf]

animals

MDPI

Table S2. Between-group variation of log10-normalized advertisement call parameters in males with and without mating success. Groups were compared using an ANCOVA with temperature as a covariate. Data are given as least square means and corresponding standard error.

**Bioacoustic parameter**	**Successful males** (N=11, 50 calls)	**Unsuccessful males** (N=35, 155 calls)	**Significance P**
Call duration [ms]	3.5914±0.0248	3.5734±0.0137	0.5322
Number of pulse groups per call	1.7517±0.0238	1.7704±0.0132	0.5011
Repetition rate of pulse groups [n per s]	1.1603±0.0047	1.1970±0.0026	**<0.00001**
Pulse group duration [ms]			
2 pulses per pulse group	1.3685±0.0126	1.3490±0.0072	0.1928
3 pulses per pulse group	1.4526±0.0069	1.4191±0.0040	**<0.00001**
4 pulses per pulse group	1.5057±0.0062	1.4748±0.0039	**0.0001**
5 pulses per pulse group	1.5677±0.0065	1.5221±0.0070	**0.0009**
Interpulse group intervals [ms]			
2 pulses per pulse group	1.6194±0.0107	1.5667±0.0061	**<0.00001**
3 pulses per pulse group	1.5728±0.0090	1.5684±0.0052	0.6761
4 pulses per pulse group	1.6640±0.0092	1.6005±0.0054	**0.0001**
5 pulses per pulse group	1.6487±0.0101	1.6792±0.0124	0.1323
Number of pulses per call	2.2428±0.0276	2.2661±0.0153	0.4681
Pulse rate [n per s]	1.6514±0.0096	1.6928±0.0053	**0.0003**
Dominant frequency [Hz]	3.1250±0.0050	3.1122±0.0027	**0.0282**
